# Closed-Loop Bowel Obstruction Secondary to Stercoroma in a Young Adolescent: A Case Report

**DOI:** 10.7759/cureus.97828

**Published:** 2025-11-26

**Authors:** Dennis Amamnkwah, Nayan Pinto, Anand Arora, Anang Pangeni, Prashant Naik

**Affiliations:** 1 General Surgery, William Harvey Hospital, East Kent Hospitals University NHS Foundation Trust, Ashford, GBR; 2 Pathology, William Harvey Hospital, East Kent Hospitals University NHS Foundation Trust, Ashford, GBR

**Keywords:** closed-loop bowel obstruction, hartmann’s procedure, megacolon, peritoneal contamination, stercoroma

## Abstract

Faecal impaction (stercoroma) is an uncommon but serious cause of large bowel obstruction (LBO) in the adolescent population. We report a rare case of a 16‑year‑old boy with longstanding constipation who developed a closed‑loop LBO secondary to a massive stercoroma. The patient presented with abdominal distension, respiratory distress, and haemodynamic instability. Imaging revealed marked colonic dilatation due to obstructing faecal matter and a competent ileo-caecal valve. Despite initial attempts at rectal evacuation, surgical intervention was required. A Hartmann’s procedure with decompression and resection of megacolon was performed successfully. This case highlights the importance of early recognition and timely surgical management of stercoroma‑related LBO in young patients.

## Introduction

Faecal impaction, characterized by the accumulation of a large, hardened mass of stool in the colon or rectum, is an uncommon but important cause of large bowel obstruction (LBO) in adolescents [1,2]. While more frequently seen in elderly or immobile patients, adolescents with risk factors such as chronic constipation or underlying motility disorders may develop severe impaction leading to bowel obstruction [3]. LBO caused by faecal impaction, also known as stercoroma, can result in serious complications, including closed-loop obstruction and bowel ischemia if not promptly recognized and treated [4]. In-hospital mortality rate for patients with faecal impaction is 8.4%, which increases to 13.5% in patients aged 85 years or older [5].

We report a rare case of stercoroma-induced LBO in an adolescent, highlighting the critical role of early surgical involvement, intraoperative strategies to reduce contamination risk, and coordinated interprofessional care. We emphasize key surgical and intraoperative considerations necessary to minimize complications and optimize outcomes in similarly severe presentations.

## Case presentation

**Figure 1:**
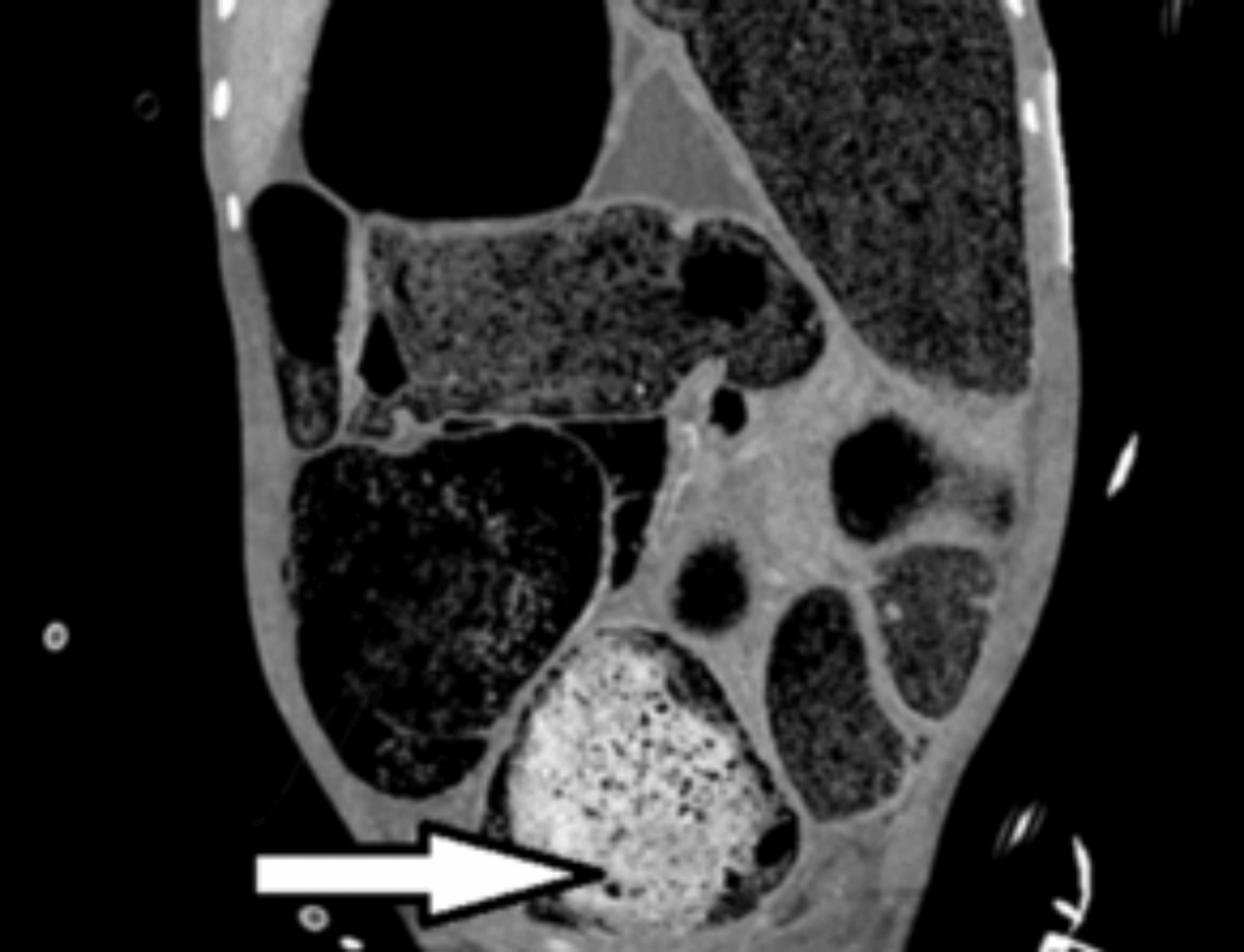
Coronal view showing a grossly dilated large bowel (caecal diameter approximately 15 cm) causing elevation of the diaphragm. A calcified stercoroma is visible in the rectum, suggesting chronicity of the condition (arrow).

**Figure 2:**
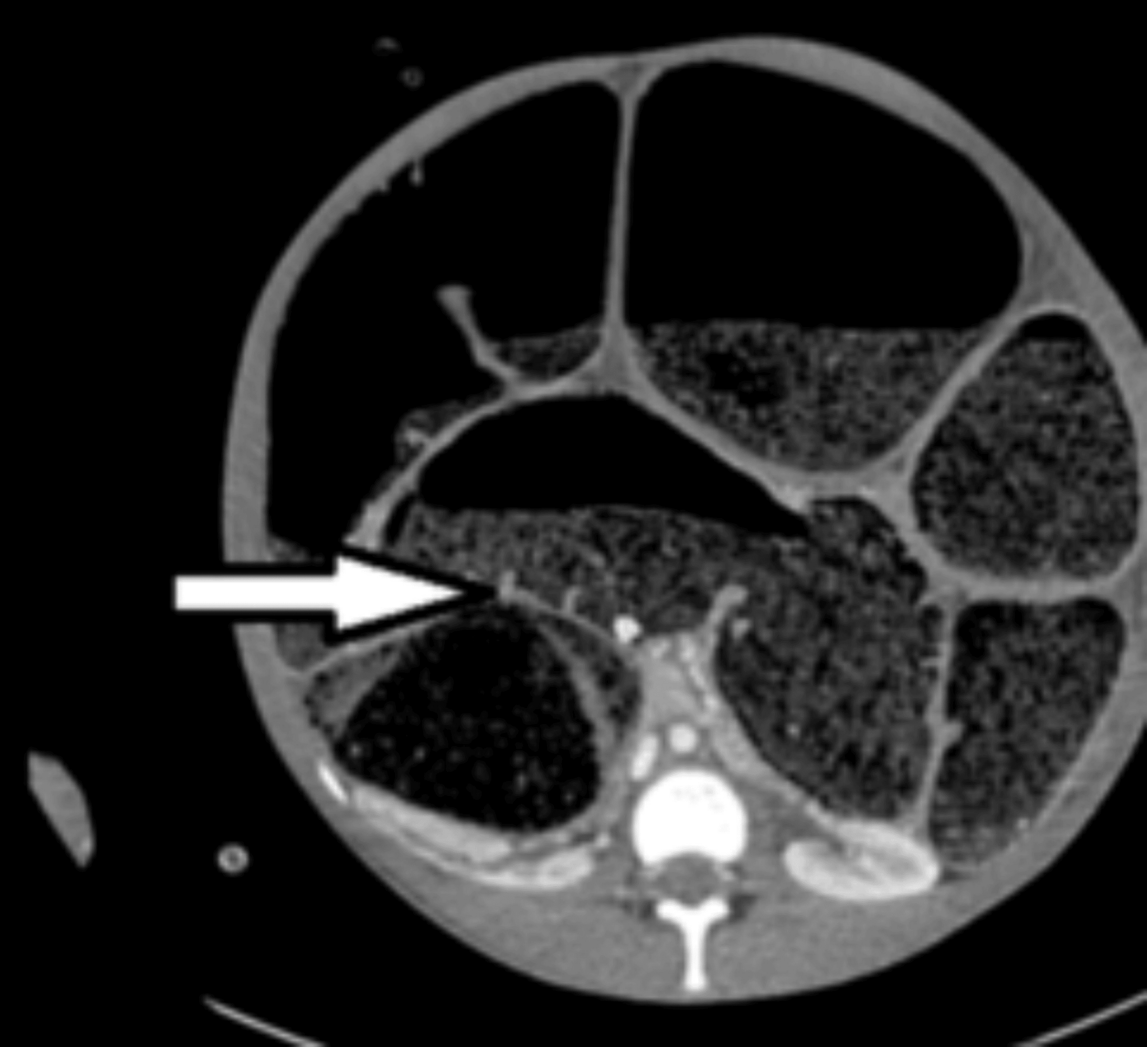
Axial view revealing a grossly distended abdomen with dilated bowel loops containing faecal matter and gas, resulting in compression of the small bowel and adjacent structures (arrow).

A 16-year-old boy, known to have chronic constipation since childhood, presented to the Accident and Emergency (A&E) Department with a closed-loop LBO with dilatation of the colon from cecum to rectum secondary to hard stercoral impaction. At presentation, he had been constipated for three days and was unwell. On initial evaluation, he was in obvious respiratory compromise due to massive abdominal distension. Vitals revealed tachycardia, tachypnoea, and hypotension, and he was drowsy and diaphoretic. Arterial blood gas (ABG) analysis revealed respiratory alkalosis. He was intubated in view of severe respiratory distress and peri-arrest. Examination of the abdomen revealed tense distension with absent bowel sounds. After initial resuscitation, he underwent a CT scan of the abdomen and pelvis, which revealed faecal impaction in the rectum, causing LBO with marked dilatation of the entire large bowel (Figure 1). There was no dilatation of small bowel loops, suggesting a competent ileo-caecal valve. There was no pneumoperitoneum, and the urinary bladder was displaced anterosuperiorly by the dilated rectum (Figure 2).

**Figure 3:**
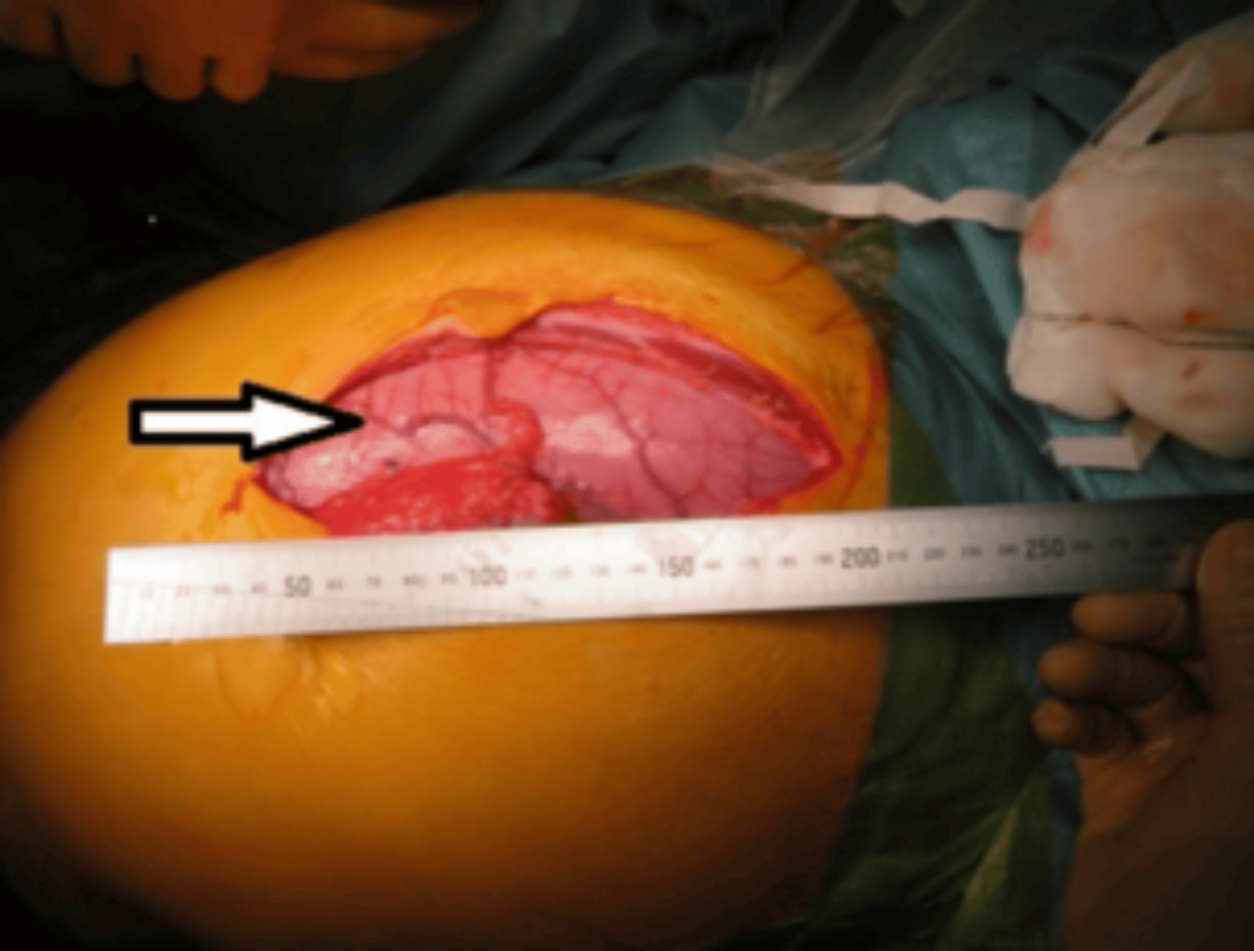
Laparotomy showing the vertical extent of caecal dilatation and abdominal distension (arrow).

**Figure 4:**
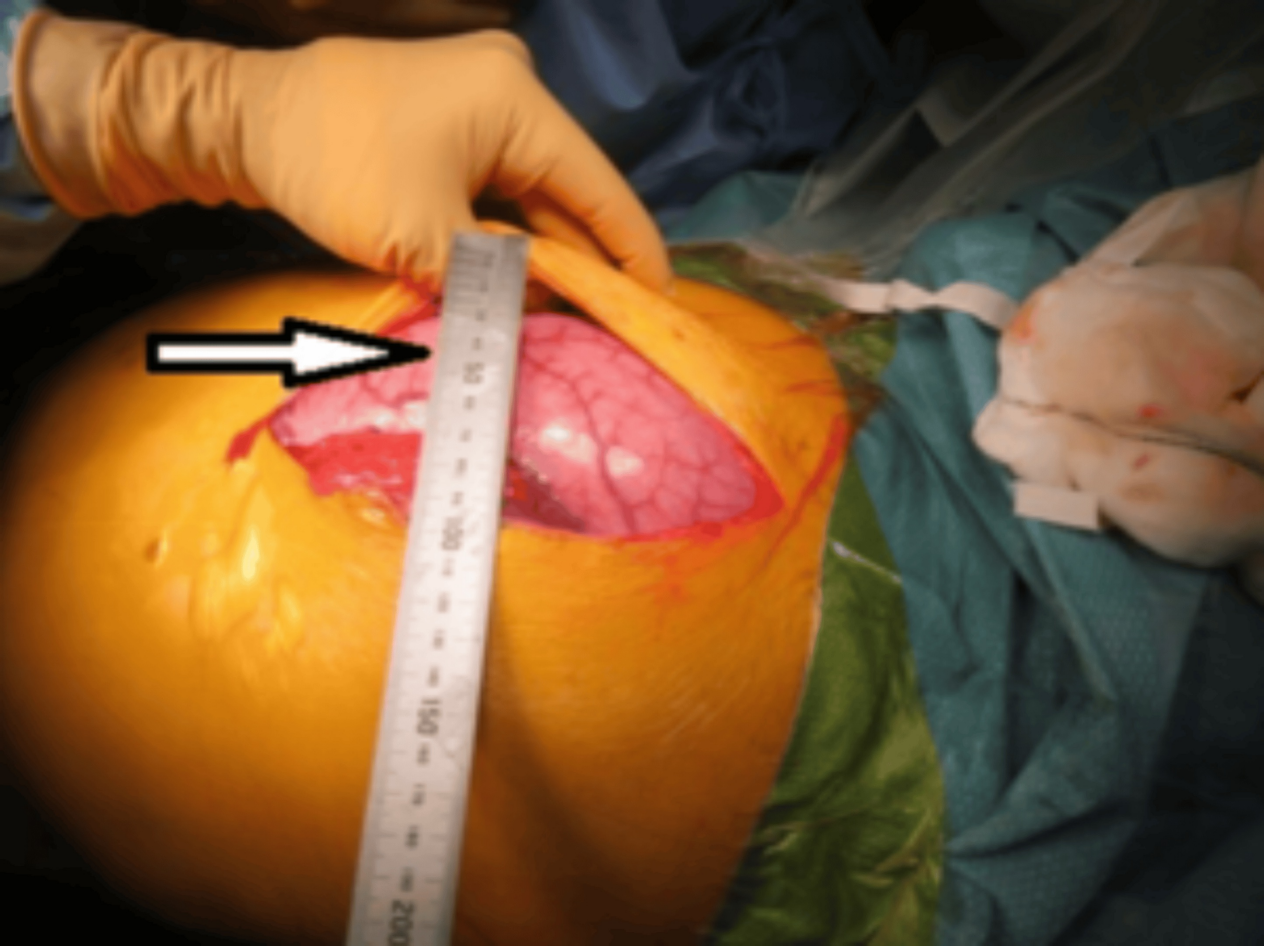
Laparotomy showing measurement of the caecal diameter in situ (arrow).

**Figure 5:**
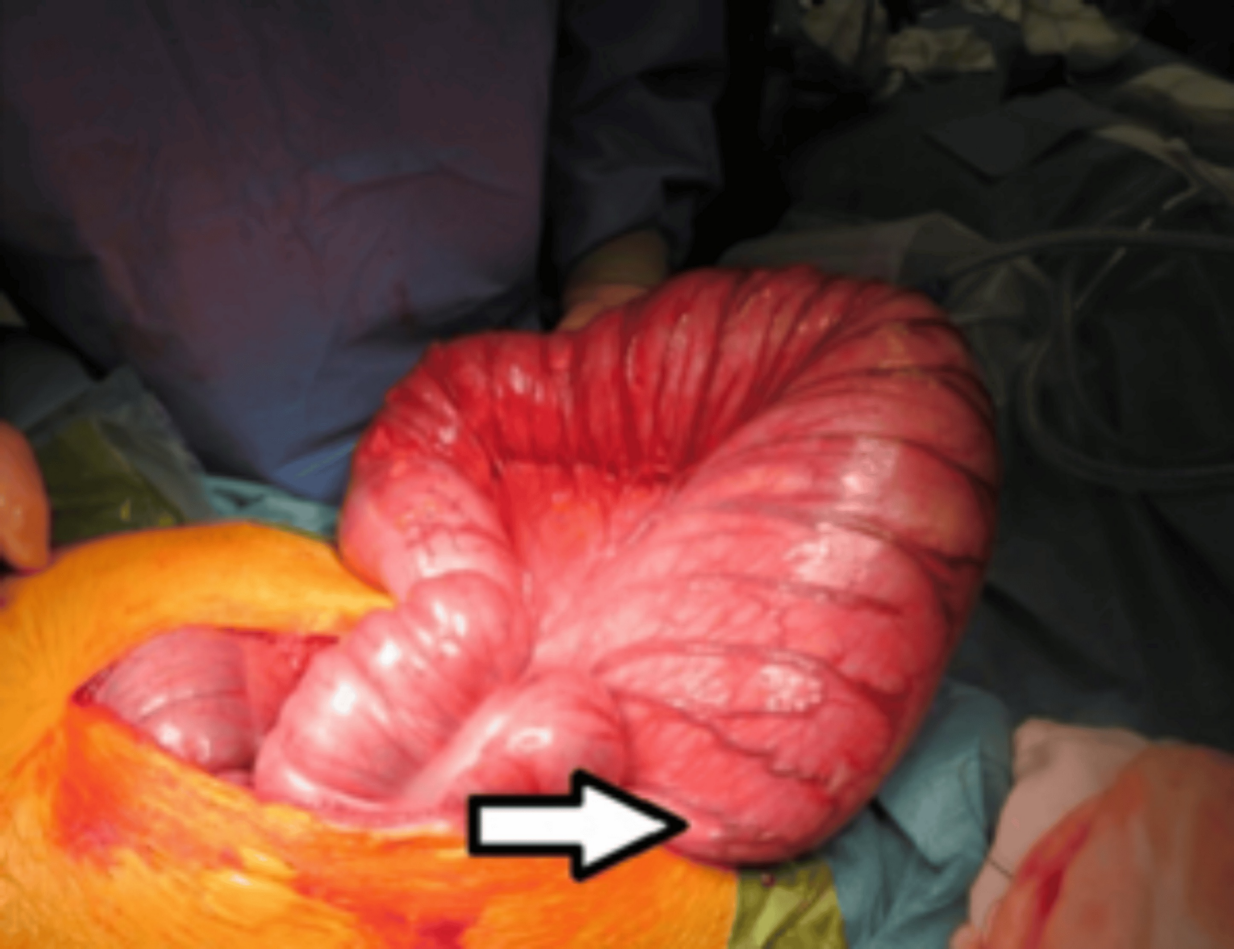
Large bowel exteriorized from the abdominal cavity just before enterotomy and suction. The underlying bowel appears healthy with no evidence of ischaemia (arrow).

The patient was positioned in a modified Lloyd-Davies position, and an attempt was made to perform a manual evacuation from the anal end to empty the rectum. Approximately one kilogram of faeces was evacuated from the ano-rectum. He then underwent an emergency laparotomy (American Society of Anesthesiologists Physical Status Classification III) (Figures 3-4), which revealed a megacolon causing a closed-loop obstruction due to a competent ileo-caecal valve (Figure 5).

**Figure 6:**
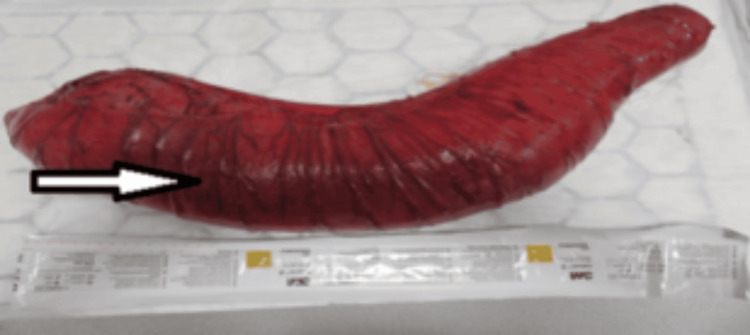
Sigmoidectomy specimen demonstrating a dilated, floppy bowel with increased and stretched vasculature (arrow).

An enterotomy was made in the sigmoid colon and decompressed till the caecum. The left hemicolon was mobilized lateral-to-medial from the proximal descending colon to the upper rectum. The rectum was then decompressed by finger-fracture of the large faecaloma inside the rectum and milking it proximally in stages, after which the upper rectum was transected with a 100 mm linear cutter and the distal stump was formed and left intrapelvic. The sigmoid colon was then transected with a linear stapler to remove a 65 cm segment of severely dilated sigmoid colon (Figure 6).

Complete decompression of the colon was achieved from the caecum to the descending colon, ensuring no peritoneal contamination during the process. The stapled end was brought out as an end colostomy (Hartmann’s procedure) in the left lumbar area. Abdominal irrigation and proctowash were then performed. On account of the staged faecal evacuation and careful surgical techniques employed to avoid peritoneal soiling, a drain was not placed in situ as abdominal irrigation was deemed sufficient.

Postoperatively, the patient was kept on ventilatory support, gradually weaned off, and extubated. He tolerated clear fluids on postoperative day 2, which were then escalated to full feeds. He made a good recovery and was discharged on postoperative day 5.

**Figure 7:**
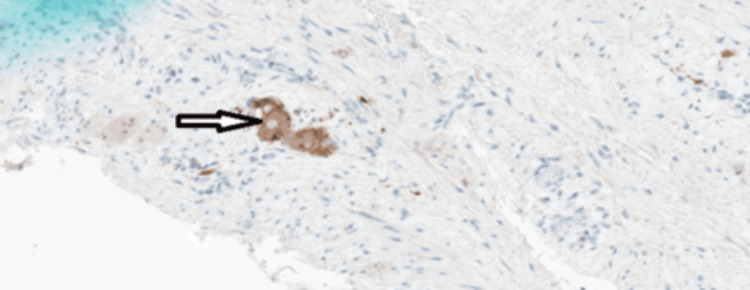
Histopathology micrograph showing myenteric plexus ganglion cells highlighted with synaptophysin (arrow).

Histopathology of the resected bowel showed no evidence of Hirschsprung’s disease or malignancy (Figure 7).

The patient remains under colorectal follow-up with plans for colostomy reversal and long-term bowel management.

## Discussion

Faecal impaction (stercoroma or faecoloma) occurs when dehydrated, compacted faecal matter accumulates, most commonly in the rectosigmoid colon. This impaction results in mechanical obstruction, colonic distension, and increased intraluminal pressure [1,3]. As pressure builds, it compromises mesenteric blood flow, leading to ischemia, pressure necrosis, and potentially perforation [4].

If the ileo-caecal valve is competent, a closed-loop obstruction may develop, in which both proximal and distal outlets are blocked [2]. This scenario is particularly dangerous as it rapidly increases pressure within the looped segment, heightening the risk of rupture during surgical handling.

Colonic dilatation may progress to megacolon, defined radiologically as caecal dilation >12 cm and sigmoid dilation >6.5 cm at the pelvic brim. In the context of stercoroma, the resultant megacolon further exacerbates ischemia, increasing the urgency for prompt surgical intervention [3]. Chronic megacolon may be congenital (Hirschsprung’s disease) or may represent end-stage refractory constipation [6].

Diagnosis of LBO secondary to stercoroma can be challenging, especially in adolescents, where such complications are unexpected. Clinical features may mimic functional constipation or nonspecific abdominal pain. In this case, imaging revealed marked colonic dilation and faecal loading - findings consistent with prior reviews identifying CT as the diagnostic modality of choice [2,3,7]. CT can demonstrate faecalomas, colonic wall thickening, and pericolonic fat stranding, confirming stercoral obstruction [3].

Initial management usually includes conservative measures such as enemas, oral laxatives, or manual disimpaction. Endoscopic disimpaction may be an option in early or uncomplicated cases, as described in a young adolescent by Thomas et al. [6]. However, when closed-loop obstruction or ischemia is suspected, surgical intervention becomes essential [7]. Preoperative preparation with adequate fluid resuscitation, broad-spectrum antibiotics, and gastric decompression can improve patient outcomes and reduce intraoperative risks.

Intraoperatively, the surgical approach was carefully adapted to minimize the risk of peritoneal contamination and subsequent complications. A staged evacuation technique was employed, in which the impacted stercoroma was gently broken down within the rectum and progressively milked proximally. Once adequate decompression was achieved, the descending colon was transected, and the stapled line was opened for complete evacuation. This was followed by a thorough rectal evacuation and a proctowash, after which the stapled rectal stump was buried securely. These modifications were essential in preventing intraoperative faecal spillage, reducing the risk of common postoperative complications such as intra‑abdominal collections, abscess formation, wound infection, and prolonged ileus - factors known to contribute significantly to morbidity, mortality, and length of hospital stay.

Postoperatively, the patient had a smooth recovery. He spent two days in intensive care and an additional five days on the surgical ward and was discharged after demonstrating stoma competence. Notably, he experienced no early postoperative complications. He remains under follow‑up in our colorectal clinic, where histological analysis of the resected specimen showed no evidence of adult‑onset Hirschsprung’s disease or other underlying pathology. Adult Hirschsprung’s disease, although rare, should always be considered in chronic constipation cases with colonic dilation, but in this instance was confidently excluded both histologically and clinically.

Because reversal of a Hartmann’s colostomy is technically demanding, many published series report a median interval of 6-18 months before restoration of continuity [8]. However, retrospective data suggest that early reversal (45-120 days) is feasible in well-selected patients and may lead to fewer postoperative complications, shorter hospital stays, and lower readmission rates [9]. That said, the decision must be individualized, especially after an emergency Hartmann’s because patient stability, adhesions, and local inflammation may preclude very early surgery. In our patient such as in any other adolescent, growth and nutrition are especially critical when planning reversal; therefore, optimization of weight, albumin, and overall nutritional status is essential before reversal.

Closure has been planned once he is optimized and provided the distal bowel is healthy, anatomical factors are favorable, slow bowel transit is improved on conservative and pharmacological management, and there are no contraindications (such as active pelvic sepsis, immunosuppression, or severe comorbidities).

This case underscores the importance of a controlled and staged approach in the operative management of faecaloma-related obstruction. Surgical options include total or subtotal colectomy, sigmoidectomy, Hartmann’s procedure, or diverting stoma creation. In this case, Hartmann’s procedure was performed, effectively relieving the obstruction and preventing perforation. During surgery, techniques such as bowel decompression, gentle manipulation, and constant suction/irrigation are essential to minimize the risk of bowel spillage and peritoneal contamination. Postoperative vigilance, continued antibiotics, and early enteral feeding contribute to optimal recovery.

## Conclusions

Intraoperatively, a staged evacuation approach minimized the risk of peritoneal contamination and postoperative sepsis. Gentle finger fracture and controlled decompression allowed for safe resection and stoma formation. Postoperative recovery was uneventful, and histology excluded Hirschsprung’s disease. This case highlights that, although rare, stercoroma-induced closed-loop obstruction can occur in adolescents, requiring early surgical involvement and a structured, multidisciplinary approach to avoid severe outcomes. It emphasizes that critical surgical considerations in emergency management, such as early recognition and careful intraoperative handling, particularly decompression and contamination control, are vital to prevent life-threatening complications.
